# Perspective: Drawing on Findings From Critical Illness to Explain Myalgic Encephalomyelitis/Chronic Fatigue Syndrome

**DOI:** 10.3389/fmed.2022.818728

**Published:** 2022-03-08

**Authors:** Dominic Stanculescu, Jonas Bergquist

**Affiliations:** ^1^Independent Researcher, Sint-Martens-Latem, Belgium; ^2^Division of Analytical Chemistry and Neurochemistry, Department of Chemistry – Biomedical Center, Uppsala University, Uppsala, Sweden; ^3^The Myalgic Encephalomyelitis/Chronic Fatigue Syndrome (ME/CFS) Collaborative Research Centre at Uppsala University, Uppsala, Sweden

**Keywords:** post-viral fatigue, hypoperfusion, endotheliopathy, gut permeability, endotoxemia, pituitary, non-thyroidal illness syndrome, myalgic encephalomyelitis/chronic fatigue syndrome (ME/CFS)

## Abstract

We propose an initial explanation for how myalgic encephalomyelitis / chronic fatigue syndrome (ME/CFS) could originate and perpetuate by drawing on findings from critical illness research. Specifically, we combine emerging findings regarding (a) hypoperfusion and endotheliopathy, and (b) intestinal injury in these illnesses with our previously published hypothesis about the role of (c) pituitary suppression, and (d) low thyroid hormone *function* associated with redox imbalance in ME/CFS. Moreover, we describe interlinkages between these pathophysiological mechanisms as well as “vicious cycles” involving cytokines and inflammation that may contribute to explain the chronic nature of these illnesses. This paper summarizes and expands on our previous publications about the relevance of findings from critical illness for ME/CFS. New knowledge on diagnostics, prognostics and treatment strategies could be gained through active collaboration between critical illness and ME/CFS researchers, which could lead to improved outcomes for both conditions.

## Introduction

Myalgic encephalomyelitis/chronic fatigue syndrome (ME/CFS) is a debilitating illness that affects millions of people worldwide (an estimated 800,000 to 2.5 million in the USA) (1, 2). Impaired function, post-exertional malaise, and unrefreshing sleep are core symptoms (1, 3, 4). At least one-quarter of ME/CFS patients are house- or bedbound at some point in their lives (1); the illness can be completely incapacitating (5). The etiology of the illness is unclear (6, 7) and peri-onset events include infection-related episodes, stressful incidents, and exposure to environmental toxins (8).

Critical illness refers to the physiological response to virtually any severe injury or infection, such as head injury, burns, cardiac surgery, SARS-CoV-2 infection and heat stroke (9). Researchers make a distinction between the *acute* phase of critical illness—in the first hours or days following severe trauma or infection; and the *chronic* or *prolonged* phase—in the case of patients who survive the acute phase but for unknown reasons do not start recovering and continue to require intensive care (10–13). Regardless of the initial injury or infection, these “chronic Intensive Care Unit (ICU) patients” experience profound muscular weakness, cognitive impairment, pain, vulnerability to infection, etc. (9, 11, 14). The treatment of *prolonged* critical illness is incomplete and remains an active area of research. Moreover, cognitive and/or physical disability can last for months or even years after treatment in ICUs (i.e., post intensive care syndrome, PICS) for as of yet unexplained reasons (15–17).

Drawing on findings from critical illness, we here propose an initial explanation for how ME/CFS could originate and perpetuate. Specifically, we combine emerging findings regarding (a) hypoperfusion and endotheliopathy, and (b) intestinal injury in these illnesses with our previously published hypothesis about the role of (c) pituitary suppression, and (d) low thyroid hormone *function* associated with redox imbalance in ME/CFS. Moreover, we describe interlinkages between these pathophysiological mechanisms as well as “vicious cycles” involving cytokines and inflammation that may contribute to explain the chronic nature of these illnesses. This explanation summarizes and expands on our previous publications about the relevance of findings from critical illness for ME/CFS (18–20) and builds on the work by Nacul et al. (21). The general lack of large high-quality ME/CFS studies (a reflection of the lack of funding in this field) poses a challenge for the assessment of overlaps between the two conditions.

## Pathophysiological Mechanisms

In the following sections we describe four central pathophysiological mechanisms in critical illness, including their relationship to inflammation. We also provide initial arguments for suggesting that similar mechanisms may underlie ME/CFS. Readers are referred to our prior publications for additional details about these mechanisms in critical illness (including heat stroke) and possible lessons for understanding ME/CFS (18–20).

### Hypoperfusion and Endotheliopathy

It has long been suggested that inadequate oxygen circulation is central to critical illness (22). Specifically, the redistribution of blood away from the splanchnic area to critical tissues is considered an adaptive androgenic response to physiological stress (23, 24). However, the resulting ischemia / reperfusion (I/R) can contribute to tissue injury driving sepsis and multi-organ dysfunction (25, 26). The relative importance of reduced blood flow, vasoconstriction (27), capillary flow disturbances (28) and impaired cellular oxygen utilization (29, 30) in driving critical illness continues to be debated.

Endothelial dysfunction appears to occur in parallel with circulation disturbances during critical illness. Probable drivers of distortions in the structure and function of endothelial lining (i.e., glycocalyx) are cytokines (31), inflammation, exposure to oxidative stress (28, 32) and/or sympatho-adrenal hyperactivation (33). Crucially, endothelial dysfunction during critical illness has been associated with altered cerebral blood flow (34, 35) and increased blood–brain barrier (BBB) permeability resulting in long-term cognitive impairment (36, 37). A leaky BBB could also contribute to increased intracranial pressure (38, 39). Finally, researchers have found that endotheliopathy and coagulation disorder bolster each other via inflammatory pathways (40). Coagulation abnormalities vary in critical illness, but coagulopathy is associated with unfavorable outcomes in prolonged critical illness (i.e., length of ICU stay and mortality) (41).

We propose that similar alterations of the vascular system in response to a physical, infectious and / or emotional stressor (i.e., physiological insult) may also contribute to explain the emergence of ME/CFS. This is consistent with recent hypotheses describing vasoconstriction in muscle and brain as a principal element of ME/CFS (42–46), and findings of cerebral hypoperfusion (47–49) and intracranial hypertension (50) in ME/CFS patients. It is also consistent with studies that have shown that endothelial function is impaired in ME/CFS (51, 52), both in large vessels and in the microcirculation (53, 54)—associated with redox imbalance (51). Finally, it is consistent with a new hypothesis for ME/CFS which suggests that endothelial senescence underpins ME/CFS by disrupting the intestinal barriers and BBBs (55), as well as with suggestions that leakage from dysfunctional blood vessels could explain many of the symptoms in ME/CFS (56).

### Intestinal Injury

Critical illness researchers have found profound intestinal alterations within hours following a physiological insult: a dramatic shift in the composition and virulence of intestinal microbes (57–59), an erosion of the mucus barrier, an increase in the permeability of the gut (i.e., “leaky gut”) (60–62), and a disruption in gut motility (63). This intestinal injury is thought to be largely a consequence of local I/R and redox imbalance resulting from splanchnic hypoperfusion (58, 61, 64–67). Indeed, studies in the field of exercise immunology have shown that even relatively low levels of splanchnic hypoperfusion during exercise result in intestinal injury (68).

Critically, this intestinal injury may lead to bacterial translocation from the gut into circulation (i.e., endotoxemia) and/or the formation of toxic gut-derived lymph (57, 60). This in turn can induce pro-inflammatory cytokines and systemic inflammation (69, 70). Moreover, changes in the intestinal microbiome or the mucus barrier may also impact the immune system directly (57). Thus, researchers have long considered the gut “the motor of critical illness” driving sepsis and distant organ dysfunction (71). Some have suggested that a self-perpetuating vicious inflammatory cycle centered around intestinal injury can hinder recovery from critical illness (61, 72).

We propose that the sequence during critical illness—from splanchnic hypoperfusion to hypoxia, redox imbalance, altered gut microbiome, intestinal injury, gut-related endotoxemia, pro-inflammatory cytokines and systemic inflammatory—may also contribute to explain the emergence of ME/CFS following a physiological insult. Our proposal is in alignment with others' findings that intestinal injury and resulting inflammation are central to ME/CFS (73–81) and consistent with findings linking the gut microbiome to inflammation (82–85) and to fatigue symptoms in ME/CFS (86). If verified, the existence of a vicious inflammatory cycle centered around intestinal injury could contribute to explain the perpetuation of ME/CFS. Post-exertional malaise—a key symptom of ME/CFS—could be the manifestation of an accentuation in intestinal injury following exertion. Moreover, the translocation of gut microbes or toxin from the intestines to the brain (55) might contribute to explain central nervous system inflammation in ME/CFS (87–89). Finally, leaky gut is also associated with auto-immunity (90, 91)—an important factor in ME/CFS pathology (92–94).

### Pituitary Suppression

Almost immediately after a physiological insult, endocrine axes experience profound alterations considered a vital response to severe stress or injury to allow for a shift in energy and resources to essential organs and repair (95–97). Whereas, in critically ill patients who begin to recover, endocrine axes essentially normalize within 28 days of illness, in cases of *prolonged* critical illness the pituitary's *pulsatile* secretion of tropic hormones (unexpectedly) remains suppressed.

Why and how this central suppression is maintained in *prolonged* critical illness continues to be debated. Inflammatory pathways likely play a role irrespective of the nature of the original injury or infection. For example, cytokines increase the abundance and affinity of glucocorticoid receptors (GR) at the level of the hypothalamus / pituitary, thereby enhancing the negative feedback loop of the hypothalamic-pituitary-adrenal (HPA) axis, and consequently suppressing pituitary release of adrenocorticotropic hormone (ACTH) (95, 98). Similarly, cytokines up-regulate deiodinase enzymes in the hypothalamus resulting in higher local levels of the *active* thyroid hormone (T3), thereby enhancing the hypothalamic-pituitary-thyroid (HPT) axis' negative feedback loop and consequently suppressing pituitary secretion of thyroid stimulating hormone (TSH) irrespective of circulating thyroid hormone concentrations (99–101). Cytokines may also suppress the release of TSH by the pituitary directly (102, 103) contributing to a virtual complete loss of *pulsatile* TSH secretion (96).

The loss of *pulsatile* pituitary secretions has important implications for the autonomic nervous system, metabolism, and the immune system. Without sufficient *pulsatile* stimulation by ACTH, adrenal glands begin to atrophy (104, 105), compromising patients' ability to cope with external stressors and permitting excessive inflammatory responses. Erratic rather than *pulsatile* pituitary production of growth hormone (GH) leads to an imbalance between catabolic and anabolic hormones, resulting in loss of muscle and bone mass, muscle weakness, and changes in glucose and fat metabolism (106–108). Finally, suppression of the HPT axis is associated with tiredness and other hypothyroid-like symptoms (109, 110).

We propose that the sequence during critical illness—from increased release of pituitary hormones during the acute phase to suppression of the pituitary gland's *pulsatile* secretion in the prolonged phase—could also contribute to explain the emergence of ME/CFS following a physiological insult. This proposal is consistent with descriptions of ME/CFS as a progression from a hypermetabolic to hypometabolic state (21). It also aligns with a recent hypothesis relating many of the symptoms in severe ME/CFS to impaired pituitary function (111). Further support for this proposal is provided by the many previous ME/CFS studies that have documented dysfunctions in the hypothalamic–pituitary–somatotropic (HPS) axis (112–114), the HPT axis (115–120), and the HPA axis (121–136)—notably associated with inflammation and oxidative & nitrosative stress (O&NS) (137–140). Strikingly, models relating the persistence of a suppressed HPA axis in ME/CFS to a change in central GRs concentrations resemble the explanations provided for pituitary suppression in critical illness (141–146). Moreover, suppression of ACTH release would explain why in a small study ME/CFS patients were found to have 50% smaller adrenals than controls (147), resembling adrenal atrophy in prolonged critical illness. However, the relationship between the pituitary's *pulsatile* secretions, physiological alterations and severity of illness—which proved revelatory in understanding *prolonged* critical illness—remains unexplored in ME/CFS.

### Low Thyroid Hormone *Function*

Peripheral mechanisms involving cytokines lead to the rapid depression of thyroid hormone activity following a severe physiological insult (148–152). This is termed “non-thyroidal illness syndrome” (NTIS), “euthyroid sick syndrome” or “low T3 syndrome” and is thought to be an adaptive response to conserve energy resources during critical illness (152–154). The mechanisms involved include alterations in the half-life of thyroid hormone in circulation (155–157); modifications in the uptake of thyroid hormone by cells (158, 159); down- and up-regulation of deiodinase enzymes that convert the thyroid hormone into active and inactive forms respectively (156, 160); and alterations in sensitivity of cells to thyroid hormones (161–163). These alterations can lead to important tissue-specific depression in thyroid hormone *function* (164, 165) which is, however, often missed altogether in clinical settings (166) because most of the alterations do not translate into changes in the blood concentrations of thyroid hormones (164, 167, 168). Indeed, the decrease in the ratio of the *active* form of thyroid hormone (T3) relative to the *inactivated* thyroid hormone (rT3) (150, 152, 169)—considered the most sensitive marker of NTIS—may be just the “tip of the iceberg” of the depressed thyroid hormone *function* in target tissues (120, 170).

While NTIS may be beneficial in the *acute* phase of critical illness, it is increasingly seen as maladaptive and hampering the recovery of patients in the case of *prolonged* critical illness (96, 101, 152, 169, 171–173). Low thyroid hormone *function* may hamper the function of organs (170) and the activity of immune cells, including natural killer cells (174–185). Immune dysfunctions might in turn explain other pathologies, such as viral reactivation observed in ICU patients (186–188). Some critical illness researchers have proposed a model that describes how NTIS is maintained by reciprocal relationships between inflammation (notably pro-inflammatory cytokines), O&NS and reduced thyroid hormone *function*, forming a “vicious cycle” (101, 173). This model can help to explain the perplexing failure to recover of some critically ill patients in ICUs that survive their initial severe illness or injury.

We propose that low thyroid hormone *function* could also contribute to explain the emergence of ME/CFS following a physiological insult. An immune-mediated loss of thyroid hormone *function* in ME/CFS has long been suspected (117). A recent study showed that the thyroid panel of ME/CFS patients resembles that of critical illness patients, including significantly lower ratio of T3 to rT3 hormones (120). Moreover, the other elements for a “vicious cycle” which researchers have suggested perpetuate a hypometabolic and inflammatory state in critical illness are also present in ME/CFS, including inflammation (140, 189), increased O&NS (190–192) and altered cytokine profiles (193, 194).

## Discussion

Hypoperfusion and endotheliopathy, intestinal injury, pituitary suppression, and low thyroid hormone *function* are each central to *prolonged* critical illness regardless of the nature of the initial severe injury or infection (101, 173, 195, 196). We propose that, similarly, these mechanisms and their reciprocal relationships with inflammation could underlie ME/CFS regardless of the nature of the peri-onset event (i.e., infection, stressful incident, exposure to environmental toxins or other) (Table 1). Moreover, the severity of ME/CFS may be a function of the strength of these mechanisms.

**Table 1:** Central pathophysiological mechanisms in prolonged critical illness, probable drivers and implications, and initial evidence suggesting similar mechanisms in ME/CFS.

**Pathophysiological mechanisms**	**In prolonged critical illness (Probable drivers and implications)**	**In ME/CFS (Initial evidence)**
Hypoperfusion	**Drivers:** • Redistribution of blood away from the splanchnic area to critical tissues (23, 24) • Reduced blood flow, vasoconstriction (27) • Capillary flow disturbances (28) • Additional: impaired cellular oxygen utilization (29, 30) **Implications:** • Ischemia / reperfusion (I/R) • Tissue injury driving sepsis and multi-organ dysfunction (25, 26)	**Initial evidence** • Vasoconstriction in muscle and brain (42–45) • Cerebral hypoperfusion (47–49) • Intracranial hypertension (50)
Endotheliopathy	**Drivers:** • Cytokines (31), Inflammation, exposure to oxidative stress (28, 32) • Sympatho-adrenal hyperactivation (33) **Implications:** • Altered cerebral blood flow (34, 35) • Increased blood–brain barrier (BBB) permeability (36, 37) • Increased intracranial pressure (38, 39). • (variable) Coagulation disorder (40)	**Initial evidence** • Impaired endothelial function (51, 52), in large vessels and microcirculation (53, 54)—associated with redox imbalance (51) • Endothelial senescence disrupting the intestinal barriers and BBBs (55) • Redox imbalance
Intestinal injury	**Drivers:** • Local I/R and redox imbalance resulting from splanchnic hypoperfusion (58, 61, 64–67) • Disruption in gut motility (63) • Shift in the composition and virulence of intestinal microbes (57–59) **Implications:** • Erosion of the mucus barrier, increase in the permeability of the gut (i.e., “leaky gut”) (60–62) • Bacterial translocation from the gut into circulation (i.e., endotoxemia) and/or the formation of toxic gut-derived lymph (57, 60) • Pro-inflammatory cytokines and systemic inflammation (69, 70) • Direct impacts on the immune system (57) • Vicious inflammatory cycle centered around intestinal injury (61, 72) • Decreased secretion of gastrointestinal hormones including ghrelin (63, 197) impacting pituitary activity	**Initial evidence** • Intestinal injury and resulting inflammation (73–81) • Altered gut microbiome linked to inflammation (82–85). • Lack of beneficial gut bacteria linked to fatigue symptoms (86) • Endothelial senescence disrupting the intestinal barriers (55) • Auto-immunity (92–94)
Suppression of *pulsatile* pituitary function	**Drivers** • Cytokines acting on abundance and affinity of glucocorticoid receptors (GR) at central level (95, 98) • Cytokines affecting deiodinase enzymes in the hypothalamus (99–101) • Direct action of cytokines on TSH release by the pituitary directly (102, 103) **Implications** • Loss of ACTH pulsatility: atrophy of adrenal glands (104, 105) compromising patients' ability to cope with external stressors and permitting excessive inflammatory responses • Loss of GH pulsatility: imbalance between catabolic and anabolic hormones, resulting in loss of muscle and bone mass, muscle weakness, and changes in glucose and fat metabolism (106–108). Alterations in deiodinase enzyme (D3) activity enabling low thyroid hormone *function* (96, 108, 198) • Loss of TSH pulsatility (109, 110)	**Initial evidence** • Progression from a hypermetabolic to hypometabolic state (21) • Impaired pituitary function (hypothesis) (111) • Dysfunctions in HPS axis (112–114), HPT axis (115–120) and HPA axis (121–136) – associated with inflammation O&NS (137–140) • Changes in central GRs concentrations (models) (141–146) • Smaller adrenals (147)
Low thyroid hormone *function*	**Drivers** • Alterations in the half-life of thyroid hormone in circulation (155–157) • Modifications in the uptake of thyroid hormone by cells (158, 159) • Down- and up-regulation of deiodinase enzymes that convert the thyroid hormone into active and inactive forms, respectively (156, 160) • Alternations in sensitivity of cells to thyroid hormones (161–163) **Implications** • Tissue-specific depression in thyroid hormone *function* (164–166) • Hampered function of organs (170) • Altered activity of immune cells, including natural killer cells (174–185) • Viral reactivation (186–188) • Vicious inflammatory cycle (101, 173)	**Initial evidence** • Immune-mediated loss of thyroid hormone *function* in ME/CFS (suspected) (117) • Significantly lower ratio of T3 to rT3 hormones (120)

**Figure 1:**
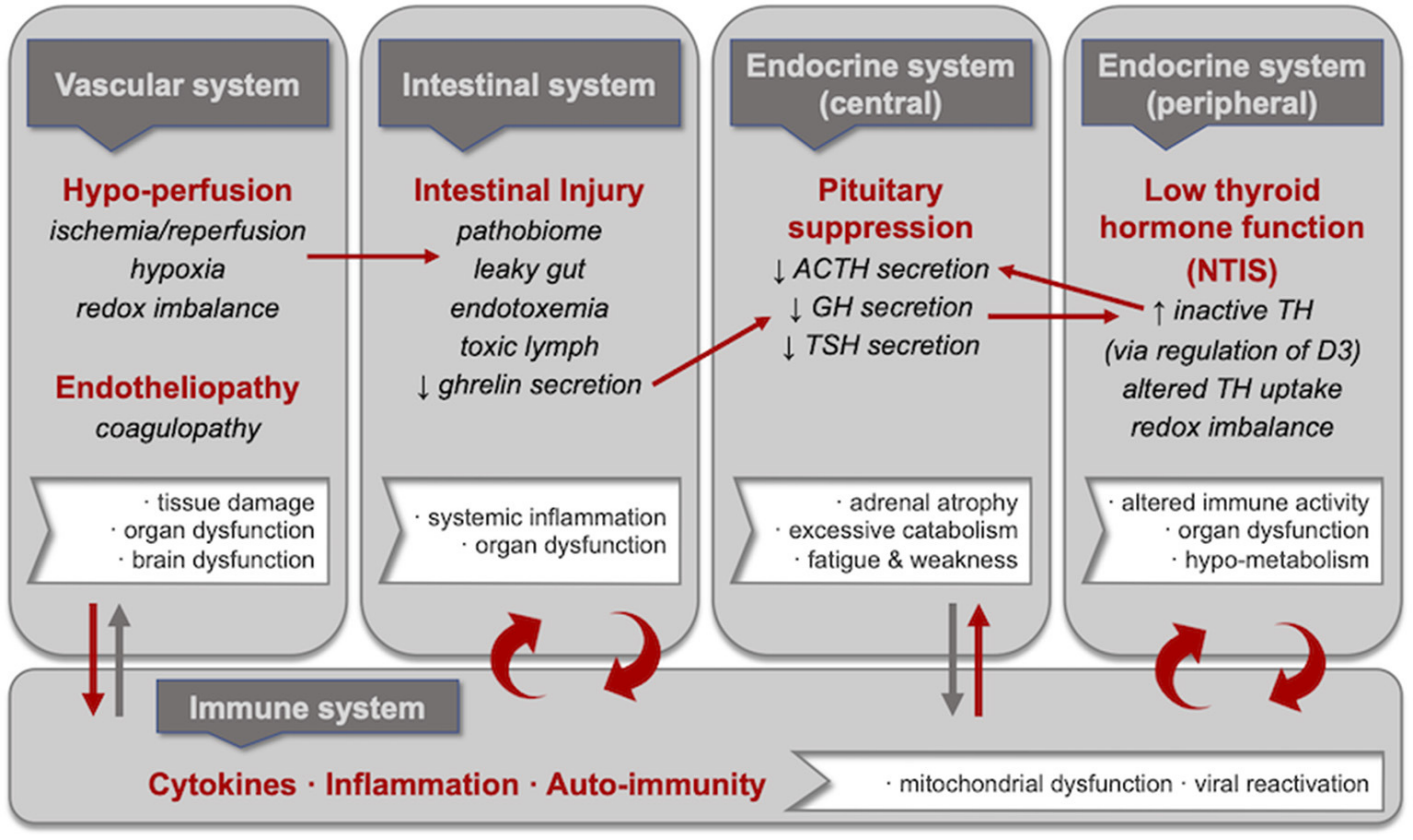
Central pathophysiological mechanisms in critical illness including selected consequences and inter-linkages. Hypoperfusion and endotheliopathy, intestinal injury, pituitary suppression, and low thyroid hormone *function* are each central to prolonged critical illness regardless of the nature of the initial severe injury or infection. These pathophysiological mechanisms are in reciprocal relationships with inflammation; specifically, researchers have proposed vicious cycles involving intestinal injury and low thyroid hormone *function*. Moreover, linkages have been described between these pathophysiological mechanisms, including (i) hypoperfusion and intestinal injury (i.e., leaky gut resulting from ischemia/reperfusion, hypoxia and redox imbalance); (ii) intestinal injury and pituitary suppression (i.e., suppressed growth hormone release resulting from reduced ghrelin secretion by the intestines); (iii) pituitary suppression and low thyroid hormone *function* (i.e., increased inactivated thyroid hormone resulting from the upregulation of D3 deiodinase as a consequence of lower growth hormone); and (iv) low thyroid hormone *function* and pituitary suppression (i.e., decreased ACTH secretion resulting from lower levels of activated thyroid hormone). We propose that these mechanisms and the linkages between them—alongside reciprocal relationships with inflammation—could also underlie ME/CFS.

However, each of these pathological mechanisms has largely been studied in isolation and rarely have the linkages between them been explored. Yet, the aggregate of these mechanisms is likely necessary to fully explain the perpetuation of critical illness—and to inform the understanding of ME/CFS (Figure 1). Additional areas for inquiry thus include the following:

### Linkages Between Intestinal Injury and Pituitary Suppression

Intestinal injury during critical illness results in decreased secretion of gastrointestinal hormones including ghrelin (63, 197). Decreased stimulation of the pituitary and hypothalamus by ghrelin during *prolonged* critical illness in turn results in lower secretion of GH by the pituitary (199). Researchers have found that the administration of an artificial ghrelin in chronic ICU patients reactivated the pulsatile secretion of GH by the pituitary and—when done in combination with thyrotropin-releasing hormones (TRH)—had beneficial metabolic effects (96, 108, 198). Similarly, the administration of ghrelin to the I/R rats “inhibited pro-inflammatory cytokine release, reduced neutrophil infiltration, ameliorated intestinal barrier dysfunction, attenuated organ injury, and improved survival” (200). The sequence between intestinal injury, ghrelin secretion and GH release by the pituitary could be particularly relevant for solving ME/CFS given that “several of the main typical symptoms in severe ME/CFS, such as fatigue, myalgia, contractility, delaying muscle recovery and function, exertional malaise, neurocognitive dysfunction, and physical disability may be related to severe GH deficiency” (111).

### Linkages Between Pituitary Suppression and Low Thyroid Hormone *Function*

There are several pathways linking the activity of the pituitary with that of thyroid hormones. Firstly, GH secreted by the pituitary co-regulates the activity of the deiodinase enzyme (D3) responsible for the conversion of thyroid hormones into inactive forms (i.e., rT3 and inactivate forms of T2) (106, 201). Researchers showed that normalization of the GH secretion in *prolonged* critically ill patients is necessary to inhibit the increase in plasma rT3 concentrations (96, 108, 198). In other words, dampened GH release by the pituitary during *prolonged* critical illness enables low thyroid hormone *function*. Secondly, the lack of stimulation of the adrenals by ACTH could (by causing an atrophy of adrenals) create the condition necessary for persistent inflammation which depresses the activity of thyroid hormones during critical illness (148–152). In other words, dampened ACTH release by the pituitary during *prolonged* critical illness might permit the vicious inflammatory cycles described above. Thirdly, there is evidence that thyroid hormone conversely also stimulates ACTH secretion (202, 203). In summary, the bi-directional relationships between the endocrine axes and thyroid hormone *function* (in addition to reciprocal relationships with inflammation) could contribute to explain the persistence of chronic ICU and ME/CFS.

### Linkages Between Low Thyroid Hormone *Function* and Endothelial Function

Upon binding to specific receptors on endothelial cells, thyroid hormones (T3 and T4) activate the endothelial nitric oxide synthase (eNOS) responsible for nitric oxide (NO) production (204), which in turn impacts vasodilation and inflammation (205–207). A further line of inquiry may thus be the role of thyroid hormone *function* in endotheliopathy in ME/CFS, including as it relates to the new finding that plasma from ME/CFS patients inhibits eNOS and NO production in endothelial cells (208). Relatedly, critical illness researchers have found that serum from patients with NTIS inhibits the uptake of thyroid hormone (209, 210); the mechanisms remain unresolved (165).

### Linkages to Mitochondrial Function

The impaired perfusion, redox imbalance, lower thyroid hormone *function* and inflammation appear to collectively affect mitochondrial activity in critical illness (via inhibition, damage, and/or decreased turnover of new mitochondrial protein) (30, 211–213). Mitochondrial activity may be similarly affected in ME/CFS (190). Some have suggested that this down-regulation of mitochondrial activity (and oxygen utilization) in critical illness may be an adaptive form of “hibernation” to protect cells from death pathways (30, 213). This suggestion echoes the hypothesis that ME/CFS is a form of “dauer” or “cell danger response” (214–216). Lower mitochondrial activity in turn affects the immune system and the gut endothelial “such that the host's immune response and physical barriers to infection are simultaneously compromised” (29).

### Relevance of Critical Illness Treatment Trials for ME/CFS

Although prolonged critical illness remains unresolved, early treatment trials—such as the reactivation of the pituitary, or interruption of the vicious inflammatory cycles centered around either gut injury or low thyroid hormone *function*—may provide therapeutic avenues for ME/CFS (19). Longitudinal studies of (spontaneous) recovery from critical illness may also give clues about prerequisites for recovery from ME/CFS. Researchers have, for example, found that “supranormal TSH precedes onset of recovery” from prolonged critical illness (96) and that metabolic rate rises > 50% above normal in the recovery phase (213).

### Commonality With Other Illnesses

Researchers have suggested commonality in the illnesses induced by physical, infectious, and / or emotional stressors (132, 217). These include heat stroke, fibromyalgia, ME/CFS, prolonged critical illness, PICS, cancer-related fatigue, post-viral fatigue, post-acute COVID-19 syndrome (PACS) and long-COVID. Specifically, it is necessary to explore whether the pathological mechanisms described above also underlie long COVID—a disease which resembles ME/CFS (218–228) and can arise even after mild COVID-19 cases.

## Conclusion

Decades of research in the field of critical illness medicine have demonstrated that in response to the stress of severe infection or injury, the vascular system, intestines, endocrine axes and thyroid hormone function experience profound alterations. Self-reinforcing interlinkages between these pathophysiological mechanisms as well as “vicious cycles” involving cytokines and inflammation may perpetuate illness irrespective of the initial severe infection or injury. Without excluding possible predisposing genetic or environmental factors, we propose that the pathological mechanisms—and the interlinkages between them—that prevent recovery of some critically ill patients may also underlie ME/CFS. This initial proposal is in line with and complements several existing hypotheses of ME/CFS pathogenesis. If this hypothesis is validated, past treatment trials for critical illness may provide avenues for a cure for ME/CFS. Certainly, given the similarities described above, active collaboration between critical illness and ME/CFS researchers could lead to improved understanding of not only both conditions, but also PICS, long-COVID, PACS, and fibromyalgia.

## Data Availability Statement

The original contributions presented in the study are included in the article/supplementary material, further inquiries can be directed to the corresponding author/s.

## Author Contributions

DS wrote the first draft of the manuscript. All authors contributed to manuscript revision, read, and approved the submitted version.

## Funding

The Open Medicine Foundation (JB) is acknowledged for support.

## Conflict of Interest

The authors declare that the research was conducted in the absence of any commercial or financial relationships that could be construed as a potential conflict of interest.

## Publisher's Note

All claims expressed in this article are solely those of the authors and do not necessarily represent those of their affiliated organizations, or those of the publisher, the editors and the reviewers. Any product that may be evaluated in this article, or claim that may be made by its manufacturer, is not guaranteed or endorsed by the publisher.

## Abbreviations

BBB: Blood–brain barrier
ACTH: Adrenocorticotropic hormone
GH: Growth hormone
GR: glucocorticoid receptors
HPA: hypothalamus-pituitary-adrenal axis: “Adreno-cortical axis”
HPS: Hypothalamic-pituitary-somatotropic axis: “Somatropic axis”
HPT: Hypothalamic-pituitary-thyroid: “Thyrotropic axis”
ICU: Intensive Care Unit
I/R: Ischemia/reperfusion
ME/CFS: Myalgic Encephalomyelitis/Chronic Fatigue Syndrome
NO: Nitrox oxide
NTIS: Non-thyroidal illness syndrome
O&NS: oxidative and nitrosative stress
PACS: Post-acute COVID-19 syndrome
PICS: Post-intensive care syndrome
TRH: Thyrotropin-releasing hormone
TSH: Thyroid stimulating hormone.
